# The effect of multidomain lifestyle intervention on health care service use and costs - secondary analyses from the Finnish Geriatric Intervention Study to Prevent Cognitive Impairment and Disability (FINGER): a randomised controlled trial

**DOI:** 10.1093/ageing/afae249

**Published:** 2024-11-22

**Authors:** Maria Sääskilahti, Jenni Kulmala, Markku Nurhonen, Jenni Lehtisalo, Markku Peltonen, Francesca Mangialasche, Tiina Laatikainen, Timo Strandberg, Riitta Antikainen, Jaakko Tuomilehto, Hilkka Soininen, Miia Kivipelto, Tiia Ngandu

**Affiliations:** Department of Public Health, Finnish Institute for Health and Welfare, P.O Box 30, FI-00271 Helsinki, Finland; Department of Public Health, Finnish Institute for Health and Welfare, P.O Box 30, FI-00271 Helsinki, Finland; Faculty of Social Sciences (Health Sciences) and Gerontology Research Center (GEREC), University of Tampere, Arvo Ylpön katu 34, 33520 Tampere, Finland; Division of Clinical Geriatrics, Center for Alzheimer Research, Department of Neurobiology, Care Sciences and Society, Karolinska Institutet, Solnavägen 1, 171 77 Solna, Sweden; Department of Public Health, Finnish Institute for Health and Welfare, P.O Box 30, FI-00271 Helsinki, Finland; Department of Public Health, Finnish Institute for Health and Welfare, P.O Box 30, FI-00271 Helsinki, Finland; Institute of Clinical Medicine, University of Eastern Finland, P.O Box 1627, Kuopio, Finland; Department of Public Health, Finnish Institute for Health and Welfare, P.O Box 30, FI-00271 Helsinki, Finland; Division of Clinical Geriatrics, Center for Alzheimer Research, Department of Neurobiology, Care Sciences and Society, Karolinska Institutet, Solnavägen 1, 171 77 Solna, Sweden; FINGERS Brain Health Institute, Karolinska vägen 37A, QA32, 171 64 Solna, Sweden; Theme Inflammation and Aging, Medical Unit Aging, Karolinska University Hospital, SE-141 86 Stockholm, Sweden; Department of Public Health, Finnish Institute for Health and Welfare, P.O Box 30, FI-00271 Helsinki, Finland; Institute of Public Health and Clinical Nutrition, University of Eastern Finland, P.O. Box 1627. FI-70211 Kuopio, Finland; Siun sote Health and Wellbeing Services County, Tikkamäentie 16, 80210 Joensuu, Finland; University of Helsinki and Helsinki University Hospital, P.O. Box 340, Helsinki, Finland; Research Unit of Population Health/Geriatrics, University of Oulu, P.O. Box 8000, Oulu, Finland; Research Unit of Population Health/Geriatrics, University of Oulu, P.O. Box 8000, Oulu, Finland; Center for Geriatrics and General Medicine, Oulu University Hospital, Pohde Wellbeing Services County of North Ostrobothnia, P.O. Box 10, FI-90029 OYS, Finland; Medical Research Center Oulu, Oulu University Hospital, P.O. Box 8000, Oulu, Finland; Department of Public Health, Finnish Institute for Health and Welfare, P.O Box 30, FI-00271 Helsinki, Finland; South Ostrobothnia Central Hospital, Hanneksenrinne 7, 60220 Seinäjoki, Finland; Department of Public Health, University of Helsinki, P.O. Box 20, 00014, Helsinki, Finland; Diabetes Research Group, King Abdulaziz University, Jeddah 22252, Saudi Arabia; Institute of Public Health and Clinical Nutrition, University of Eastern Finland, P.O. Box 1627. FI-70211 Kuopio, Finland; Department of Neurology, Kuopio University Hospital, P.O. Box 100, Kuopio, Finland; Department of Public Health, Finnish Institute for Health and Welfare, P.O Box 30, FI-00271 Helsinki, Finland; Division of Clinical Geriatrics, Center for Alzheimer Research, Department of Neurobiology, Care Sciences and Society, Karolinska Institutet, Solnavägen 1, 171 77 Solna, Sweden; FINGERS Brain Health Institute, Karolinska vägen 37A, QA32, 171 64 Solna, Sweden; Theme Inflammation and Aging, Medical Unit Aging, Karolinska University Hospital, SE-141 86 Stockholm, Sweden; The Ageing Epidemiology Research Unit, School of Public Health, Imperial College London, London W6 8RP, UK; Department of Public Health, Finnish Institute for Health and Welfare, P.O Box 30, FI-00271 Helsinki, Finland; Division of Clinical Geriatrics, Center for Alzheimer Research, Department of Neurobiology, Care Sciences and Society, Karolinska Institutet, Solnavägen 1, 171 77 Solna, Sweden; Institute of Public Health and Clinical Nutrition, University of Eastern Finland, P.O. Box 1627. FI-70211 Kuopio, Finland

**Keywords:** older people, multidomain lifestyle intervention, health care service use, cost, register

## Abstract

**Background:**

The Finnish multidomain lifestyle intervention study to prevent cognitive impairment and disability (FINGER, N = 1259), a randomised controlled trial had beneficial effects on morbidity in older people, but to what extent such a lifestyle intervention may affect the use of health care services and their costs especially in long term are unknown.

**Objective:**

This study investigated the effect of a two-year FINGER multidomain intervention on health care service use during the 8-year follow-up. The costs of service use were also evaluated.

**Methods:**

Health care service use obtained from national health care registers (days of inpatient hospital stay and long-term care, number of visits to emergency services, hospital as outpatient, home care, primary care physician and primary care nurse) was analysed among participants of the FINGER. Trial targeted community-dwelling people aged 60–77 years at risk for cognitive impairment, who were randomly allocated to the multidomain intervention or control group. Costs were evaluated as the mean costs of services used.

**Results:**

There were no significant differences in total health care costs between the intervention and control groups. The participants in the intervention group, however, had a lower use of the hospital inpatient care (RR 0.73, 95% CI 0.54–1.00) and emergency services (RR 0.83, 95% CI 0.70–0.97) than those in the control group. Hospital inpatient care was lower especially among men. The use of other types of health care services did not differ between the groups. The costs of health care service use without including long-term care were lower in the intervention group (RR 0.81, 95% CI 0.68–0.99).

**Conclusions:**

The FINGER intervention has a potential to reduce the need for the inpatient hospital care and emergency visits and associated costs, especially among men.

## Key Points

- Studies on association of multidomain lifestyle interventions with health care service use with long-term follow-up are scarce.
- We found that FINGER intervention had beneficial effects on health care service use and costs in 8-year follow-up.
- FINGER intervention was associated with reduced use of hospital inpatient care and emergency services especially among men.
- Future studies should investigate these associations also in other populations and with an even longer follow-up.

## Introduction

Health spending increases along with ageing, and people aged 65 and over account for around 35–45% of the total health spending worldwide [1–4]. While older people are nowadays both cognitively and physically healthier than before [5–7], the increase in the number of older adults still leads to increase in the need for health care services in the future [8, 9].

Chronic conditions, such as cardiovascular diseases, diabetes and mental disorders, are the main reasons for health care service use worldwide [10]. Dementia, cardiovascular diseases and musculoskeletal disorders are the leading causes for hospitalisation and long-term care in old age in Finland [11, 12]. Healthy lifestyle plays a pivotal role in maintaining health and functioning with ageing, and the effects of lifestyle interventions on reduction of risk of chronic conditions as well as multimorbidity are relatively widely known [13–20]. In addition to the beneficial effects in chronic disease prevention, multidomain interventions are suggested to be cost-effective [21–24], but the influence of lifestyle interventions on the longer-term subsequent health care use and related costs among older people has not been widely studied.

The Finnish Geriatric Intervention Study to Prevent Cognitive Impairment and Disability (FINGER) was the first large-scale randomised controlled trial showing that targeting simultaneously several lifestyle habits and cardiovascular risk factors in an older population at risk of cognitive decline led to improvements in cognitive capacity, decreased risk of chronic diseases, better health-related quality of life, and maintenance of mobility and daily functioning [25–29]. The intervention was also potentially cost-effective [21]. By linking the trial participants’ information with data from national health care registers, the aim of this study was to assess the long-term effects of the lifestyle intervention on health care service use and costs involved.

## Materials and methods

### Participants

FINGER, a 2-year multidomain intervention study started in 2009 (ClinicalTrials.gov NCT01041989), included 1259 independently living older persons from six cities and their surrounding areas in Finland. The FINGER study protocol, recruitment of the participants, baseline characteristics and results on primary outcome have been reported in detail previously [25, 30, 31]. The inclusion criteria were age of 60–77 years at the start of the study, a Cardiovascular Risk Factors, Ageing and Dementia Risk Score of 6 points or higher, and the cognitive performance at the mean level or slightly lower than expected for age according to Finnish population norms tested with the Consortium to Establish a Registry for Alzheimer’s Disease neuropsychological battery [30]. Exclusion criteria were previously diagnosed or suspected dementia and disorders affecting safe engagement in the intervention (e.g. malignant disease, major depression, severe loss of vision or hearing, or symptomatic cardiovascular disease) and coincident participation in another intervention trial. This article is reported according to the CONSORT guideline.

### FINGER intervention

Participants were randomly assigned into the group receiving intensive multidomain intervention or regular health advice group (control) in a 1:1 ratio. Computer-generated allocation was done by study nurses in blocks of four (two individuals randomly allocated to each group) at each study site after baseline assessments. The intervention protocol and components have been described in detail previously [25, 31]. Briefly, the intervention included simultaneous nutritional counselling, physical activity intervention, cognitive training, social activities and vascular risk monitoring and management. Blinding was pursued as much as possible in lifestyle intervention. The nutritional component included three individual sessions and seven to nine group sessions conducted by study nutritionists. The physical activity component was guided by physiotherapists at the gym and consisted of individually tailored programs for progressive muscle strength training (1–3 times per week) and mainly independent aerobic exercise (2–5 times per week). Cognitive training included 10 group sessions led by psychologist and independent computer-based training at home or at study site (2–3 times per week, two six-month periods). Social activities were stimulated through the numerous group meetings. Management of metabolic and vascular risk factors consisted of regular visits to the study nurse (at 3, 9 and 18 months) and physician (at 3, 6 and 12 months) including evaluation of anthropometric measures, laboratory tests and cardiovascular and metabolic conditions, and advice to their management. Both the intervention and the control group visited the study nurse four times (baseline, 6, 12 and 24 months) and the study physician at baseline and 24 months during the active study period for measurements and physical examination. In addition, at baseline the study nurse provided both groups with general information and advice on maintaining a healthy lifestyle.

The active intervention lasted for two years for each participant (during the years 2009–2014), followed by a light maintenance intervention during 2016–2018 with text messages on healthy lifestyles (tips on healthy diet and physical, cognitive and social activities were sent weekly to the intervention group participants). Follow-up examinations for both groups took place at ~5 (during 2015–2016) and 7 (during 2017–2018) years after the baseline.

The FINGER study was approved by the coordinating ethics committee of the Hospital District of Helsinki and Uusimaa (HUS/1204/2017). The participants gave written informed consent before enrolment in the study including also consent for linking the national health register data to the clinical trial data.

### Outcome measures

The primary outcome of the FINGER intervention was cognitive performance [25, 30], which was measured with an extended version of the neuropsychological test battery. Health care service use was one of the predefined secondary outcomes of the study [30].

Data on health care service use from the FINGER baseline visit (conducted between 2009–2011) until the end of December 2018 (average follow-up time 8.02; time period referred as 2009–2018 in the following sections) were obtained from three Finnish national health care registers: Care Register for Health Care, Register of Primary Health Care Visits and Care Register for Social Welfare. The Care Register for Health Care includes data on patients discharged from public hospitals (covers most hospitals) in Finland since 1969. The register also includes information on day surgeries and outpatient visits at specialised health care facilities in hospitals dating back to 1994. The Register of Primary Health Care Visits includes data from primary health care visits (all the public and part of the private health care) since 2011. It also includes home care visits. The Care Register for Social Welfare includes data from patients/clients discharged from or living at institutional care and round-the-clock housing services since 1995. First, an ~8-year period corresponding roughly to the time span from trial baseline to the last follow-up visits was analysed. For sensitivity analyses, also a longer follow-up from the baseline until the end of June 2021 (average follow-up time 10.17 years; time periods referred as 2009–2021 in the following sections) was obtained to investigate how long the potential effect of the intervention on the health care service use may last. The extended period included also the possible effects of the COVID-19 on the provision and use of services (combination of both reduced access and demand due to lock-down measures and increased use due to the infection). We also conducted sensitivity analyses for shorter time periods, 2009–2012 and 2009–2015, to see when the changes in service use and costs actually took place.

In this study, the outcomes retrieved from the registers were days spent in hospital and long-term care (short- and long-term stays in different round-the-clock services), and the number of emergency visits, hospital outpatient visits, home care (home nursing/care services) visits, primary care physician visits and primary care nurse visits. Health care service costs were determined as their mean unit costs according to the national report of unit costs of health and social care in Finland in 2017 [32]. In this study, costs are presented as the costs of health care services without long-term care and as total costs of all studied health care services. The FINGER intervention was independent of the regular health service, and the physician and nurse visits related to the intervention were not recorded in the routine national health registers.

### Background information

Medical history was assessed through a standardised questionnaire filled in by study physicians after interviewing the participants. The study nurses conducted measurements of MMSE, blood pressure, anthropometry and took blood samples for glucose and lipids. Participants filled in questionnaires related to several other study parameters.

### Statistical analyses

The sample size calculation for the FINGER trial was based on the primary cognitive outcome [30]. Health care service use was calculated as the average number of visits or average number of days in care per person-year. The costs of health care service use were calculated as the annual average costs of health care service use per participant by the randomisation group. Follow-up time started at the date of randomisation to the trial and ended either at death or at the given end date (2018). Intervention and control groups were compared via the rate ratio (RR) or via the difference in the averages in the two groups. Differences in health care service use and costs during the average follow-up time were calculated by multiplying the differences in annual average use or cost by 8.02 years. Due to the skewness of the distributions of most outcome variables, 95% confidence intervals were evaluated with nonparametric bootstrap applying the bias-adjusted, accelerated approach [33] with 20 000 replications. We also fitted negative binomial regression models adjusted for sex and age at enrolment. In assessing differences between the intervention and control groups, the results agreed with those obtained by comparing the unadjusted average service use among the control and intervention groups separately by sex. For conciseness and ease of interpretation, we have only reported unadjusted results. Percentage of the participants who visited different types of health care services at least once during the follow-up was also assessed. Sensitivity analyses for different follow-up periods (2009–2012, 2009–2015, 2009–2021) and excluding persons who died during the follow-up were conducted using the same methods as in original analysis. The average follow-up times were 2.33 years, 5.24 years and 10.17 years. Statistical analyses were performed using R (version 4.3.2) [34] using the boot package [35].

## Results

### Population characteristics

A total of 1259 participants were randomly allocated to the study groups: 631 to the intervention group and 628 to the control group (Appendix 1). Selected baseline characteristics of the participants by group are presented in Table 1. Mean age of the participants was 69.4 years at baseline, 53.3% of them were men, and mean education was 10 years. Participants in the intervention and control groups did not differ from each other in terms of baseline demographic and health-related characteristics. Of the 1259 cohort members, 128 died (60 in the intervention group and 68 in the control group) during the years 2009–2018 and additional 95 (49 and 46, respectively) died in years 2019–2021. Of all who died, 149 were men and 74 women.

**Table 1:** Characteristics of the participants at baseline according to intervention allocation

	All (n = 1259)	Intervention (n = 631)	Control (n = 628)	P-value^a^
Age (years), mean (SD)	69.4 (4.7)	69.5 (4.7)	69.2 (4.7)	0.27
Education (years), mean (SD)	10.0 (3.4)	10.0 (3.5)	10.0 (3.4)	0.92
Women, n (%)	587 (46.6)	286 (45.3)	301 (47.9)	0.35
Married or cohabiting, n (%)	932 (74.1)	459 (72.9)	473 (75.3)	0.32
Fasting plasma glucose, mean (SD)	6.1 (0.9)	6.1 (0.8)	6.1 (1.0)	0.99
Serum total cholesterol, mean (SD)	5.2 (1.0)	5.2 (1.0)	5.1 (1.0)	0.90
Systolic blood pressure, mean (SD)	140.1 (16.2)	140.2 (16.6)	140.0 (15.7)	0.79
Diastolic blood pressure, mean (SD)	80.3 (9.5)	80.5 (9.7)	80.2 (9.3)	0.53
Body mass index, mean (SD)	28.2 (4.7)	28.3 (4.5)	28.1 (4.9)	0.46
Smokers, n (%)	114 (9.4)	64 (10.6)	50 (8.3)	0.17
Hypertension, n (%)	647 (51.7)	324 (51.6)	323 (51.8)	0.93
Cardiac insufficiency, n (%)	35 (2.8)	18 (2.9)	17 (2.7)	0.89
Angina pectoris, n (%)	77 (6.2)	44 (7.0)	33 (5.3)	0.21
Asthma, n (%)	106 (8.5)	55 (8.7)	51 (8.2)	0.72
Rheumatoid arthritis, n (%)	30 (2.4)	11 (1.8)	19 (3.0)	0.14
Cerebrovascular disease, n (%)	22 (1.8)	10 (1.6)	12 (1.9)	0.65
Diabetes, n (%)	168 (13.4)	87 (13.8)	81 (13.0)	0.66
Depression, n (%)	80 (6.4)	36 (5.7)	44 (7.1)	0.32
Hypercholesterolemia, n (%)	839 (67.2)	410 (65.4)	429 (69.0)	0.18
15D^b^, mean (SD)	0.9 (0.1)	0.9 (0.1)	0.9 (0.1)	0.63
Zung depression score^c^, mean (SD)	33.9 (7.5)	33.9 (7.8)	33.9 (7.2)	1.00
ADL^d^, mean (SD)	18.1 (2.6)	18.2 (2.9)	18.1 (2.4)	0.31
MMSE^e^, mean (SD)	26.7 (2.1)	26.7 (2.1)	26.7 (2.1)	0.60

^a^Comparison between intervention and control groups; t test for continuous variables and Pearson’s Chi-Square test for categorised variables.

^b^Health-related quality of life; scale 0–1 with higher number indicating less problems; range 0.58–1 in this population.

^c^Scale 20–80 with higher number indicating presence of more depressive symptoms; range 20–63.

^d^Activities of daily living; include basic activities of daily living and instrumental activities of daily living; scale 17 (no difficulties) - 85 (total dependence); range 17–46.

^e^Mini-mental state examination; scale 0–30 with higher number indicating better cognitive performance; range 20–30.

### Use of health care services

Of the 1259 participants, 99.4% had used at least one of the studied health care services during the 8-year follow-up. Almost all participants had visited primary care physician (96%), nurse (95%), and specialised outpatient care (94%) (Table 2). Of the participants, 69% had had an emergency visit, 66% had stayed in hospital, 34% received home care and 6% had stayed at a long-term care facility. During the extended follow-up 2009–2021, all the percentages were slightly larger (Appendix 2).

**Table 2:** Percentage of participants using health care services at least once during the 8-year follow-up (2009–2018)

	Whole group	Intervention	Control
Hospital inpatient stay, % (CI)	65.7 (63.0–68.2)	62.9 (59.1–66.7)	68.5 (64.8–72.0)
Emergency visit, % (CI)	68.8 (66.1–71.2)	67.5 (63.9–71.2)	70.1 (66.3–73.5)
Hospital outpatient visit, % (CI)	93.6 (92.1–94.8)	92.7 (90.4–94.5)	94.6 (92.5–96.2)
Physician visit in primary health care, % (CI)	96.1 (94.8–97.0)	95.7 (93.9–97.1)	96.5 (94.8–97.7)
Nurse visit in primary health care, % (CI)	94.7 (93.2–95.7)	95.4 (93.6–96.9)	93.9 (91.9–95.6)
Home care visit, % (CI)	34.0 (31.3–36.5)	32.6 (29.0–36.4)	35.4 (31.7–39.1)
Long-term care, % (CI)	6.4 (5.0–7.8)	7.0 (5.2–9.2)	5.7 (4.1–7.7)

**Figure 1:**
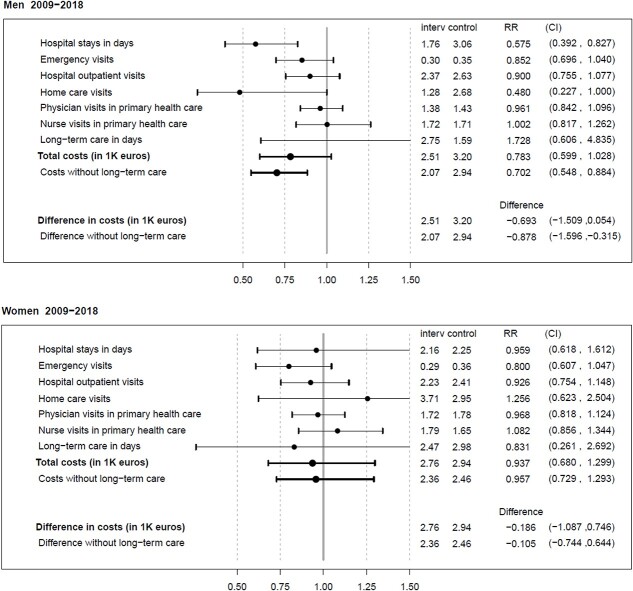
Average annual health care service use in days, number of visits, or thousand euros (interv/control) and rate ratios (RRs) with 95% CI for the intervention group vs. control in health care service use and costs among men and women during the 8-year follow-up (2009–2018). In addition, for costs, differences in thousand euros are given.

There was a difference between the intervention and control group in the number of days spent in hospital (1.94 vs. 2.67 average annual days per participant in the intervention group and control group, respectively; RR 0.73, 95% CI 0.54–1.00) resulting in 5.8 fewer in-hospital days in the intervention group during the 8-year follow-up (Table 3). In addition, the participants in the intervention group had fewer emergency visits (0.29 vs. 0.36 average annual visits per participant; RR 0.83, CI 0.70–0.97). There were no other statistically significant differences in health care use by group. Among women, no statistically significant group differences existed in any type of health care service use (Fig. 1), among men; however, the intervention group participants had less hospital days than the control group participants (RR 0.58, 95% CI 0.39–0.83) (Fig. 1).

**Table 3:** Comparison between the groups in use and costs of health care services per participant during the 8-year follow-up period (2009–2018)

	Intervention group, units per year	Control group, units per year	RR (CI) (Intervention/ control)	Difference per follow-up (intervention-control)	Unit cost, €
**Health care service use**					
Hospital stays in days, mean (CI)	1.94 (1.59–2.52)	2.67 (2.20–3.33)	0.73 (0.54–1.00)	−5.83 days	556/day
Emergency visits, mean (CI)	0.29 (0.26–0.33)	0.36 (0.32–0.40)	0.83 (0.70–0.97)	−0.50 visits	322/visit
Hospital outpatient visits, mean (CI)	2.30 (2.09–2.55)	2.52 (2.30–2.77)	0.91 (0.80–1.05)	−1.75 visits	323/visit
Physician visits in primary health care, mean (CI)	1.54 (1.43–1.65)	1.60 (1.49–1.75)	0.96 (0.86–1.06)	−0.54 visits	83/visit
Nurse visits in primary health care, mean (CI)	1.75 (1.58–1.98)	1.68 (1.52–1.90)	1.04 (0.89–1.21)	0.52 visits	40/visit
Home care visits, mean (CI)	2.39 (1.68–3.40)	2.81 (2.00–3.97)	0.85 (0.51–1.40)	−3.39 visits	35/visit
Long-term care in days, mean (CI)	2.62 (1.59–4.21)	2.26 (1.31–3.82)	1.16 (0.54–2.47)	2.90 days	160/day
**Total costs, €, mean (CI)**	2619 (2275–3078)	3073 (2681–3579)	0.85 (0.69–1.05)	−3640€	
**Costs without long-term care, €, mean (CI)**	2200 (1952–2553)	2712 (2402–3117)	0.81 (0.68–0.99)	−4110€	

In sensitivity analyses (with 10-year follow-up), the differences between the intervention and control groups in health care service use were relatively similar than those during the 8-year follow-up but they were no longer statistically significant in the whole population (Appendix 3). While there were no group differences among women (Appendix 4), among men, the intervention group participants stayed fewer days in hospital than those in the control group (RR 0.69, 95% CI 0.47–0.96) (Appendix 4). When we excluded persons who died during the follow-up, no significant differences between the groups were observed (Appendix 5 and 6). Additional sensitivity analyses showed that during a follow-up there were differences in hospital outpatient visits (during 2-year follow-up) and homecare visits (2- and 5-year follow-up) among men in favour of the intervention group (Appendix 7).

### Costs

The total costs of health care service use, without including the costs of long-term care, were lower in the intervention group (Table 3) (RR 0.81, 95% CI 0.68–0.99) resulting in 4110€ cumulative cost saving during 8 years per intervention group participant compared to the participants in the control group. Among men, health care service costs were lower in the intervention than the control group (RR 0.70, 95% CI 0.55–0.88) (Fig. 1). The group differences were no longer statistically significant when long-term care costs were included. Among women, there were no group differences in costs (Fig. 1). In sensitivity analyses with 10-year data, health care service costs without long-term care were lower in the intervention than the control group (RR 0.78, 95% CI 0.62–0.97) among men (Appendix 4). There were no other group differences (Appendix 3, Appendix 4).

## Discussion

We reported the health care service use and costs among community-dwelling people aged 60 years and over who were at increased risk for cognitive impairment and were included in the multidomain lifestyle intervention. The people in the intervention group had less in-hospital days and emergency visits. This difference was seen in hospital stays only among men, whereas the effect on emergency visits was similar in both sexes. The health care service costs were lower in the intervention group, especially among men.

Our study provides unique data of the effect of multidomain lifestyle intervention on health care service use, as long register-based follow-ups are scarce [36]. There are some studies reporting short-term effects of multidomain or single-domain lifestyle interventions on health care service use and costs, with some of the studies being register-based and in others, health care service use is self-reported by participants [22–24, 37, 38]. With a longer follow-up, there are some cohort studies investigating the association of lifestyles and health care service use but these studies lack the intervention aspect [39].

Our results indicate that lifestyle intervention was effective in preventing or delaying conditions that require hospital care. Comorbidity, severity of diseases, functional decline, respiratory diseases and cardiovascular diseases are the main risk factors associated with hospitalisation in older people [40]. In general, women have more disabling chronic diseases, but in men more severe and fatal conditions such as cardiovascular diseases occur [41] and these may lead to increased use of hospital care after the emergency visits which may partly explain also the sex differences observed. In our sensitivity analyses, we found that hospital care use was highest among people who died during the follow-up which supports this hypothesis. Multidomain lifestyle interventions have a role in reduction of cardiovascular disease risk especially in the high-risk populations [18]. It has been previously shown that the FINGER intervention decreased the risk for cardiovascular events [27] and lowered the risk of new chronic diseases [26]; these benefits may explain a part of the reduced need for hospital care among men. Physical activity interventions have been shown to reduce the risk of falls and related care needs [37, 42, 43], and although the incidence of falls was not studied in the FINGER, this association may be one possible explanation in decreased hospital care need. In general, the FINGER intervention was able to maintain mobility and daily functioning [29] which may reduce falls and injuries in older people and thus decrease the need for hospital care.

The intervention group had a lower need for emergency health care visits. Emergency visits are related to hospital stays as, in many cases, emergency care precedes hospital stay [44]. However, fewer emergency visits in the intervention group were observed in both sexes whereas the effect of intervention on hospital stays was seen only in men. We did not investigate the reasons for hospital stays or emergency visits but focused on the service use only. The previously identified main reasons for emergency visits in older people are cardiovascular problems, mental health issues, musculoskeletal, neurological, respiratory and abdominal conditions [45]. The beneficial effects of FINGER intervention on some of these conditions may explain a part of the decrease in emergency visits.

Hospitalisation and emergency visits are increased with ageing [40, 46]. As the populations all around the world are getting older, multidomain lifestyle interventions may have important impact on morbidity and health care service needs. For example, in Finland, health care services suffer from labour shortage, and actions that reduce the need for those services are demanded [47].

The FINGER intervention had no effects on the use of primary care services, i.e. primary care nurse and physician visits. During the intervention visits for the management of cardiovascular diseases, the study physician did not prescribe treatments but guided the participants to contact primary care if needed. According to our findings, this did not result in an increased use for primary care services. Also, the control group received feedback for their risk factor levels measured during the outcome assessment visits, which may have also resulted in increased contacts with health care also among them. On the other hand, the participants visited study nurses and physician several times during the intervention period and received results of examinations during the intervention. This may have decreased the need for examinations and visits in primary health care. The multidomain lifestyle intervention had no effect either on long-term or home care need. However, there is much uncertainty due to the strong overdispersion in these outcomes as also seen with the wide confidence intervals.

The FINGER intervention was associated with savings in health spending which partially result from the decrease in hospital inpatient care. It is the most expensive health care service, thus the reduction in its use is also mirrored in reduction in costs. The measure of health care service costs in this study is crude. It is an average estimate of the costs in a specific service type and not the real costs of the service use. In addition, the costs lack the medication costs that are over a tenth of total health spending in Finland [48]. However, our results suggest that the multidomain lifestyle intervention has a potential to reduce the costs of health care services by decreasing the need for expensive health care services, such as in-hospital care. In addition, the FINGER intervention has been shown to have a potential to be cost-effective by decreasing the risk for cognitive impairment [21].

It looks like the benefits of the 2-year intervention on health care service use and costs were slightly diluted during the longer 10-year follow-up as compared to the 8-year follow-up. In the Look AHEAD study in people with type 2 diabetes, health care service use and costs during the 10-year lifestyle intervention and the 3-year post-intervention follow-up were examined. The lifestyle intervention reduced hospitalisations, hospital days and number of medications during the intervention period, resulting also in reduction of costs [49]. During the 3-year post-intervention follow-up, those differences diminished, and the researchers’ concluded that the intervention should be sustained for achieving effects on long-term health care spending [36]. Our study, however, suggests that multidomain lifestyle intervention may be effective in reducing health care service use and costs also after the active intervention period, even if the effect of intervention is diluted over extended time period. Dilution of the intervention effect in the long run is understandable as along with ageing people are having increasing numbers of health problems resulting in an increasing need for health care services [2, 40, 46]. The dilution of the results seen in this study may also partially be consequence of the COVID-19 pandemic and related lock-down that reduced health care service use especially during the year 2020 [50, 51].

The strength of this study is carefully designed randomised controlled trial setting with comprehensive register data comprising all public health care service use and some of the private health care service use (all day surgeries and hospital days) in Finland. In Finland, 80% of all health care expenditure is covered by public sector [52], and the data included in the national registries cover most of the hospitals, primary care units, occupational health care, home care service providers, and long-term care units. However, it is a weakness that the national health register data lacks information on private primary care visits, care given outside the borders of Finland, and family caregiving. The other weakness of the register data is that practices to record data may change over time. In addition, sex difference in intervention effect on hospital days we found warrant further studies to investigate e.g. sex differences in causes of death.

## Conclusions

Multidomain lifestyle intervention has potential to reduce the need for hospital inpatient and emergency care and related costs. Based on our findings, the awareness of benefits of healthy lifestyle should be more emphasised in health care contacts, since changing the lifestyle into healthier direction have wide-ranging effects on health, functioning and need of care. This is the first study investigating the long-term effects of a lifestyle intervention on health care services and cost. The issue should be studied also in other trials and settings and with an even longer follow-up.

## Supplementary Material

aa-24-0616-File002_afae249
