# Supplementary material for: The effect of multidomain lifestyle intervention on health care service use and costs - secondary analyses from the Finnish Geriatric Intervention Study to Prevent Cognitive Impairment and Disability (FINGER): a randomised controlled trial
Source: Age Ageing. 2024 Nov 22;53(11):afae249. doi: 10.1093/ageing/afae249 (PMC11584201; doi:10.1093/ageing/afae249)
Supplement: aa-24-0616-File002_afae249 [file aa-24-0616-file002_afae249.docx]

**The effect of multidomain lifestyle intervention on health care service use and costs – secondary analyses from The Finnish Geriatric Intervention Study to Prevent Cognitive Impairment and Disability (FINGER) randomised controlled trial**

Appendices:

Appendix 1. Flow chart.

Appendix 2. Percentage of participants using health care services at least once during the 10-year follow-up (2009-2021).

Appendix 3. Comparison between the groups in use and costs of health care services per participant during the 10-year follow-up period (2009-2021).

Appendix 4. Average annual health care service use in days, number of visits, or thousand euros (interv/control) and rate ratios (RRs) with 95% CI for the intervention group vs. control in health care service use and costs among men and women during the 10-year follow-up (2009-2021). In addition, for costs, differences in thousand euros are given.

Appendix 5. Average annual health care service use in days, number of visits, or thousand euros (interv/control) and rate ratios (RRs) with 95% CI for the intervention group vs. control among men and women during the 8-year follow-up (2009-2018) excluding persons who died (i.e. w/o deaths in the title of figures). In addition, for costs, differences in thousand euros are given.

Appendix 6. Average annual health care service use in days, number of visits, or thousand euros (interv/control) and rate ratios (RRs) with 95% CI for the intervention group vs. control among men and women during the 10-year follow-up (2009-2021) excluding persons who died (i.e. w/o deaths in the title of figures). In addition, for costs, differences in thousand euros are given.

Appendix 7. Average health care service use per year separately for 4 time periods. Statistically significant differences (*p*=0.05) are indicated by an asterisk. Some CIs are not available due to sparseness of nonzero values.


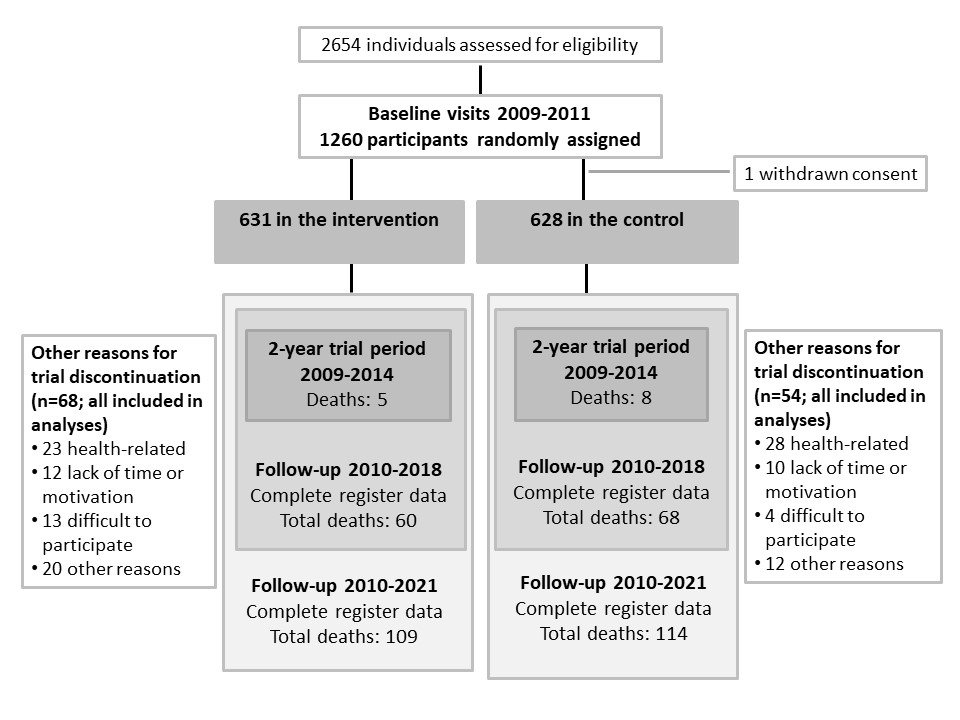


Appendix 1. Flow chart.

Appendix 2. Percentage of participants using health care services at least once during the 10-year follow-up (2009-2021).

|  | **Whole group** | **Intervention** | **Control** |
| --- | --- | --- | --- |
| Hospital inpatient stay, % (CI) | 70.5 (67.8-72.8) | 68.1 (64.3-71.6) | 72.8 (69.1-76.2) |
| Emergency visit, % (CI) | 71.9 (69.3-74.2) | 70.8 (67.1-74.2) | 72.9 (69.3-76.3) |
| Hospital outpatient visit, % (CI) | 95.0 (93.6-96.0) | 94.1 (92.1-95.8) | 95.9 (94.1-97.2) |
| Physician visit in primary health care, % (CI) | 97.1 (95.9-97.9) | 97.0 (95.4-98.1) | 97.1 (95.6-98.2) |
| Nurse visit in primary health care, % (CI) | 97.2 (96.1-97.9) | 97.5 (96.0-98.5) | 97.0 (95.4-98.1) |
| Home care visit, % (CI) | 42.4 (39.6-45.1) | 42.3 (38.5-46.2) | 42.5 (38.7-46.4) |
| Long-term care, % (CI) | 10.7 (9.1-12.5) | 11.7 (9.4-14.4) | 9.7 (7.6-12.3) |

Appendix 3. Comparison between the groups in use and costs of health care services per participant during the 10-year follow-up period (2009-2021).

|  | **Intervention group, units per year** | **Control group, units per year** | **RR (CI)** (Intervention/  control) | **Difference per follow-up** (intervention-control) | **Unit cost, €** |
| --- | --- | --- | --- | --- | --- |
| **Health care service use** |  |  |  |  |  |
| Hospital stays in days, mean (CI) | 1.95 (1.63-2.43) | 2.42 (2.03-2.95) | 0.81 (0.62-1.06) | -4.66 days | 556€/day |
| Emergency visits, mean (CI) | 0.28 (0.25-0.31) | 0.33 (0.29-0.37) | 0.85 (0.72-1.00) | -0.49 visits | 322€/visit |
| Hospital outpatient visits, mean (CI) | 2.12 (1.94-2.34) | 2.31 (2.11-2.54) | 0.92 (0.80-1.05) | -1.86 visits | 323€/visit |
| Physician visits in primary health care, mean (CI) | 1.54 (1.44-1.65) | 1.59 (1.49-1.72) | 0.97 (0.88-1.07) | -0.44 visits | 83€/visit |
| Nurse visits in primary health care, mean (CI) | 1.77 (1.61-1.98) | 1.72 (1.57-1.92) | 1.03 (0.89-1.19) | 0.46 visits | 40€/visit |
| Home care visits, mean (CI) | 4.28 (3.33-5.48) | 4.23 (3.25-5.47) | 1.01 (0.70-1.45) | 0.47 visits | 35€/visit |
| Long-term care in days, mean (CI) | 3.73 (2.55-5.41) | 3.60 (2.40-5.29) | 1.04 (0.59-1.82) | 1.27 days | 160€/day |
| **Total costs, €, mean (CI)** | 2805 (2457-3244) | 3120 (2736-3590) | 0.90 (0.74-1.09) | -3150 € |  |
| **Costs without long-term care, €, mean (CI)** | 2209 (1982-2514) | 2544 (2277-2880) | 0.87 (0.74-1.03) | -3350 € |  |


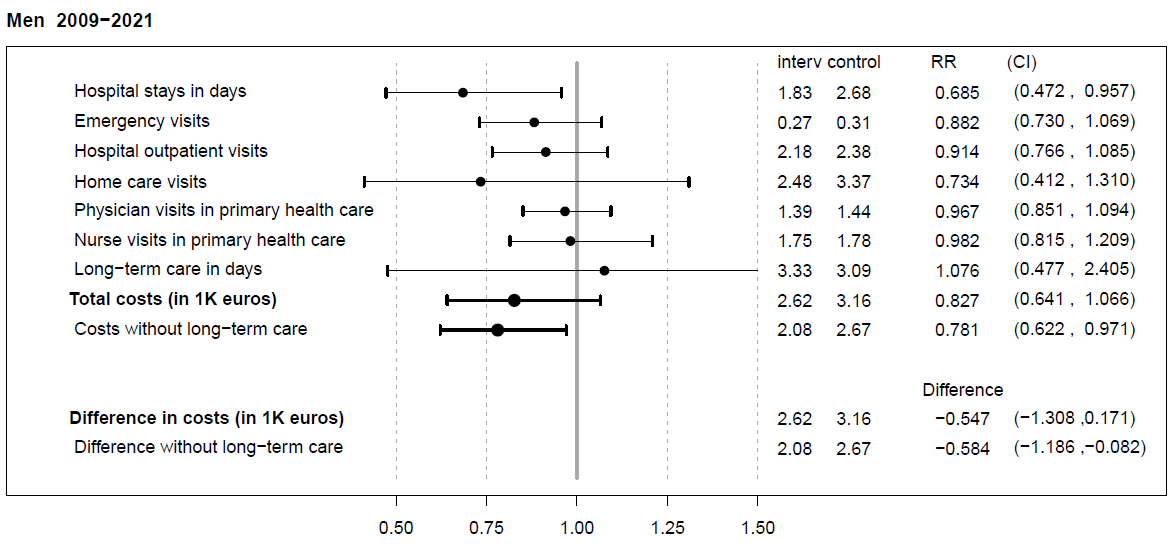


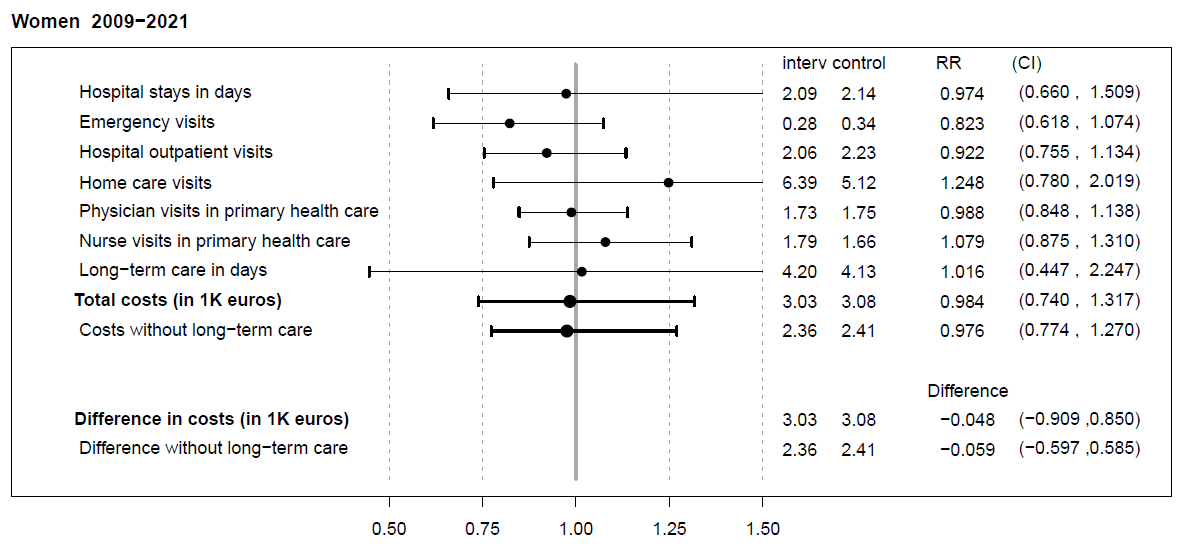


Appendix 4. Average annual health care service use in days, number of visits, or thousand euros (interv/control) and rate ratios (RRs) with 95% CI for the intervention group vs. control in health care service use and costs among men and women during the 10-year follow-up (2009-2021). In addition, for costs, differences in thousand euros are given.


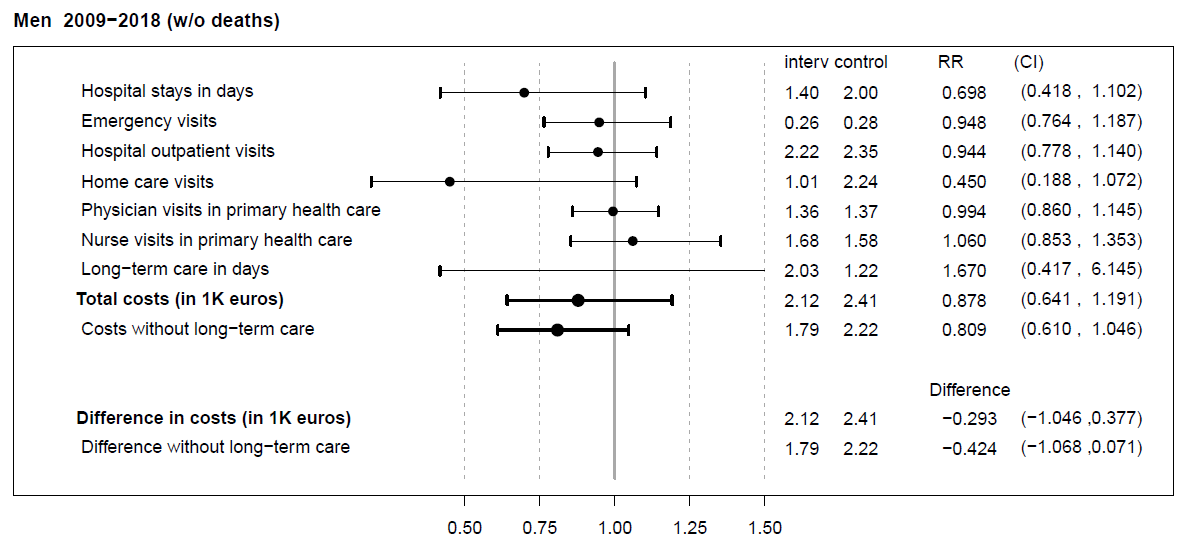


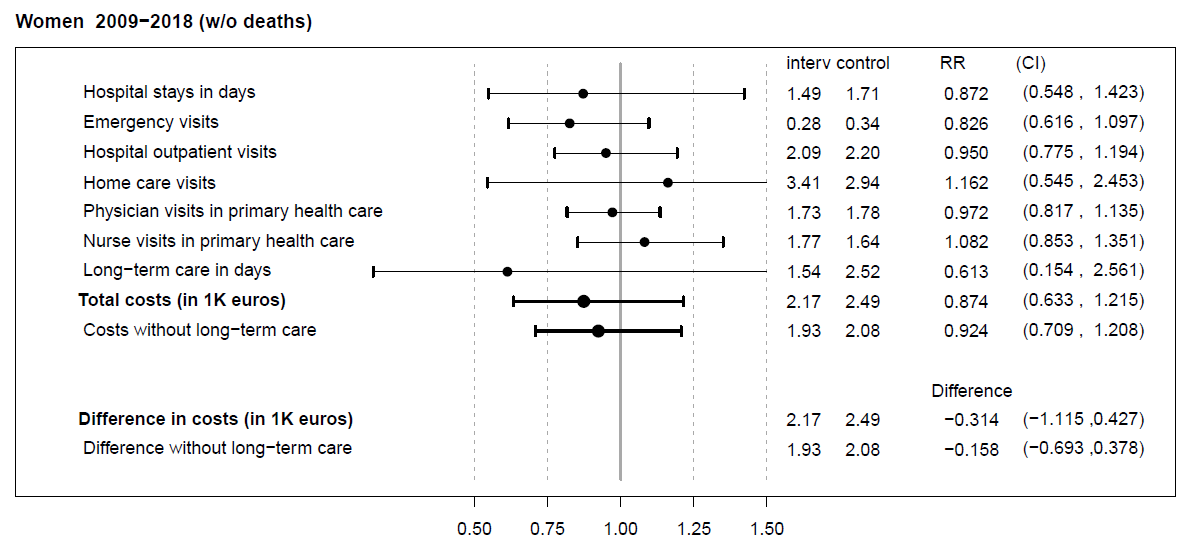


Appendix 5. Average annual health care service use in days, number of visits, or thousand euros (interv/control) and rate ratios (RRs) with 95% CI for the intervention group vs. control among men and women during the 8-year follow-up (2009-2018) excluding persons who died (i.e. w/o deaths in the title of figures). In addition, for costs, differences in thousand euros are given.


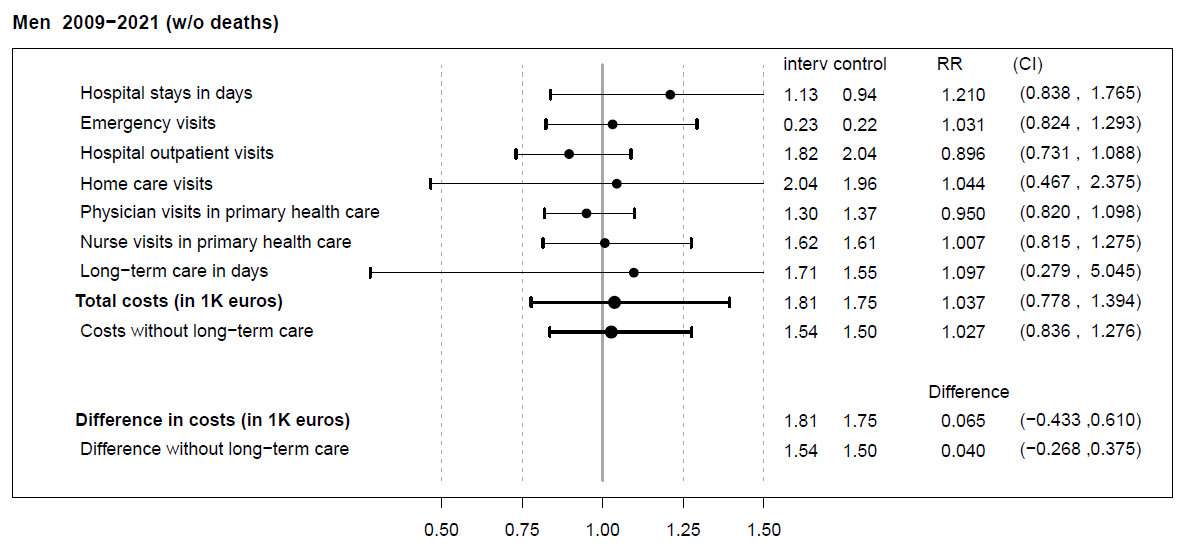


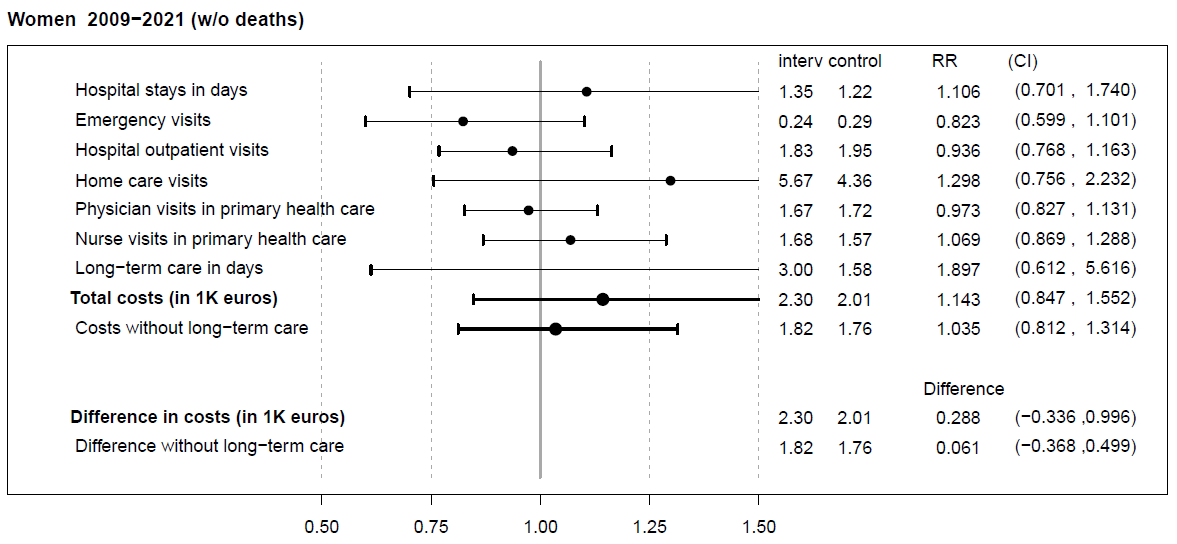


Appendix 6. Average annual health care service use in days, number of visits, or thousand euros (interv/control) and rate ratios (RRs) with 95% CI for the intervention group vs. control among men and women during the 10-year follow-up (2009-2021) excluding persons who died (i.e. w/o deaths in the title of figures). In addition, for costs, differences in thousand euros are given.


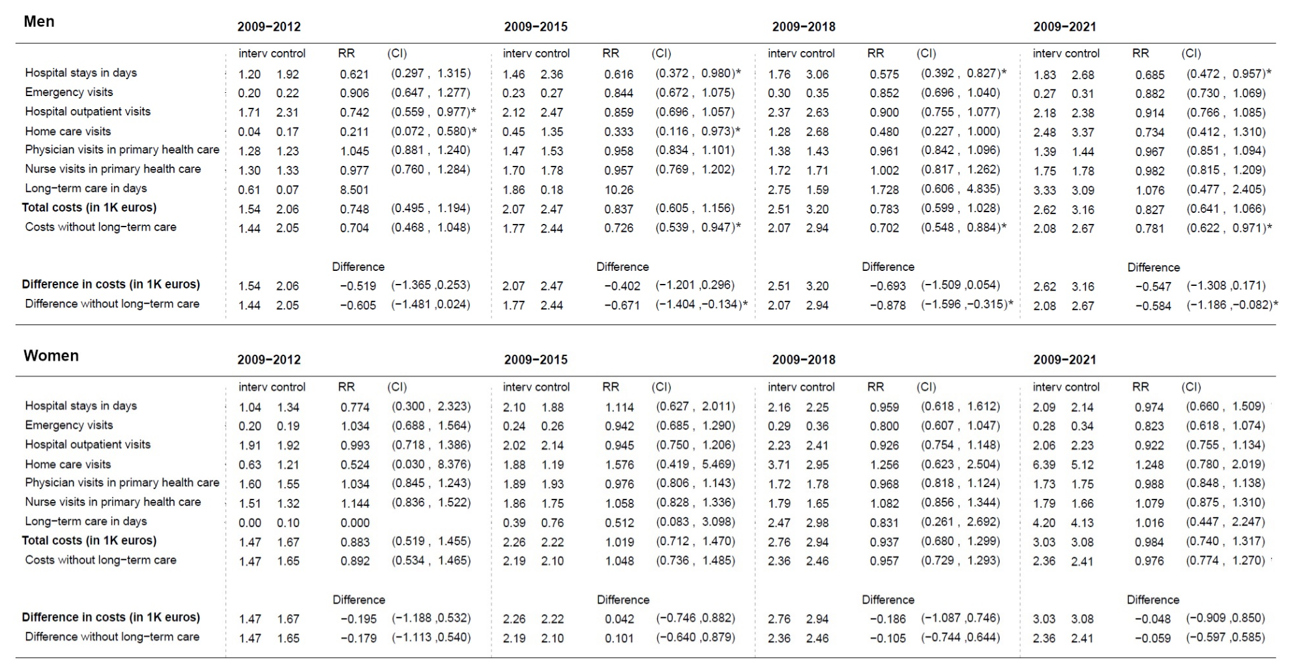


Appendix 7. Average health care service use per year separately for 4 time periods. Statistically significant differences (*p*=0.05) are indicated by an asterisk. Some CIs are not available due to sparseness of nonzero values.
